# Trauma magnitude of the meta-epyphyseal cancellous affects the incidence of deep vein thrombosis. A prospective cohort study on the dynamic of Collagen I, Collagen IV, Tissue factor, P-Selectin and Nitric Oxide in the thrombus formation following hip and knee surgeries

**DOI:** 10.1016/j.amsu.2021.102190

**Published:** 2021-02-23

**Authors:** Franky Hartono, Irawan Yusuf, Budhianto Suhadi, Andi Fachruddin, Yohanes Augustinus

**Affiliations:** a Consultant Hip and Knee Orthopaedic and Traumatology, Department of Surgery, Faculty of Medicine, University of Pelita Harapan, Tangerang, Indonesia; b Department of Physiology, Faculty of Medicine, University of Hasanuddin, Makassar, Indonesia; c Department of Bioethics, Faculty of Medicine, University of Pelita Harapan, Tangerang, Indonesia; d Division of Hematology-Medical Oncology, Department of Internal Medicine, University of Hasanuddin, Makassar, Indonesia; e Department of Orthopaedic and Traumatology, Pantai Indah Kapuk Hospital, Jakarta, Indonesia

**Keywords:** DVT, Hip and knee surgery, Meta-epiphyseal cancellous, Pro-thrombogenic biomarker, Anti-thrombogenic biomarker, Collagen I, Nitric oxide

## Abstract

**Introduction:**

The purpose of this study was to analyze the traumatization degree of meta-epiphyseal cancellous of hip and knee joints in major orthopedic surgery that affects the incident of deep vein thrombosis (DVT) event through the dynamics expression of pro-thrombogenic biomarkers (Collagen I, Collagen IV, Tissue Factor, P-selectin) and anti-thrombogenic (Nitric Oxide).

**Methods:**

In this cohort prospective study, there were sixty-nine (69) subjects that were divided into three (3) groups, with twenty-three (23) subjects that were treated with total arthroplasty (TA), twenty-two (22) subjects were treated with hemiarthroplasty (HA), twenty-four (24) subjects were treated with open reduction internal fixation (ORIF). Subjects from May 2010 to September 2011 who met the inclusion criteria were included in this study. All patients were treated without thromboprophylaxis. Blood samples were taken in three different periods, before surgery, 72 h, and 144 h after surgery, for examination of pro-thrombogenic biomarkers (Collagen I, Collagen IV, Tissue Factor, P-selectin) and anti-thrombogenic (Nitric Oxide), which are the components involved in the hemostasis.

**Results:**

DVTs were proven by venography (or Doppler ultrasound in 8 cases) done at 144 h after the surgeries. Eighteen (18) subjects had DVT (26.1%), with ten (10) subjects from the TA group (43.5%), five (5) subjects from the HA group (22.7%), and three (3) subjects from ORIF groups (12.5) %). The risk for experiencing DVT on TA is 3.5 times more than the ORIF group, while in HA group is 2.1 times more than ORIF group. The role of biomarker levels on DVT incidence was found in Col I (p < 0.1) and NO (p < 0.05) at 72 h after surgery.

**Conclusion:**

This research confirms that trauma magnitude of the meta-epiphyseal cancellous of hip and knee joints in major orthopedic surgery influences the incidence of DVTs, through the elevation of Col I and NO. An estimated 72 h after surgery is a useful period to examine these biomarkers to help predict the diagnose of DVT. The involvement of the other biomarkers studied (Col IV, TF, and Ps) could not be proven. Future studies are needed to evaluate other biomarkers in the complex process of hemostasis to establish the diagnose of DVT.

## Highlights

• Most major hip and knee surgery caused DVT included in three groups according to the degree of traumatization, namely one elective group for osteoarthrosis hip and knee as total arthroplasty (TA group), two non-elective groups for fracture at the femoral head as hemiarthroplasty (HA group) and fracture at femoral trochanter as internal fixation (ORIF group)
• Five biomarkers used to represent the dynamic component of pro- and anti-thrombogenic in the model of the hemostasis and inflammation process.
• Incidence of DVTs influenced by the trauma magnitude of the meta-epiphyseal cancellous bone through the elevation of Col I and NO at 72 h after surgery.
• The involvement of Col IV, TF and Ps have not yet been proven.

## 1 Introduction

Deep Vein Thrombosis (DVT) following major hip and knee orthopedic surgery is a condition in which there is a total or partial blockage by blood clots in the segments of lower extremities veins, e.g. the Tibial vein, Fibularis vein, Popliteal vein, Femoral vein, and Iliofemoral vein. DVT is characterized by venous hypertension, swelling of the legs, and damage to venous valves which can progress to pulmonary embolism (PE) [1].

DVT is a process of balance between thrombogenic stimulation processes that form thrombus and various protective processes that dissolve it [2]. Besides this hemostasis and fibrinolytic pathway, and inflammation process also play a big role in this thrombus formation [3]. This multifactorial process occurs through Virchow's triad, which consists of blood hypercoagulability, venous blood flow stasis, and injury to the endothelial lining of blood vessels. This pathomechanism can explain why the incidence of DVT in orthopedics (75–80%) and general surgeries (20–25%) have a higher rate than in non-operative cases or internal medicine which is 17% only [4]. The most common occurrence of DVT is found after surgery in the meta-epiphyseal area of the hip and knee joints [4].

According to the “The seventh ACCP Consensus Guidelines”, hip or knee joint replacement surgery and hip fracture repair surgery are risk factors that have the highest incidence of DVT [5]. This occurs because the intraoperative hemostasis activation and the consequences inflammation process in orthopedic are much greater than in non-bone surgery [1,6,7]. The application of Virchow's triad theory to orthopedic patients starts when the patient is immobilized due to illness, injury, anesthesia, or surgery. 1) The venous stasis increases the risk of developing DVT before, during, and after surgery [8]. Risk factors such as cardiac dysfunction, obesity, and disorders of blood vessel pumps can also aggravate stasis [9]. 2) The endothelial injury occurs through the process of destruction of muscle tissues, blood vessels, and bones through the action of muscle retractions, joints rotation, drilling, and grinding of the bones, and also the use of cement and implants in the cancellous surface of femur and knee. 3) The hypercoagulability condition is often due to acquired disorders, such as old age, obesity, elderly women, cancer, hypertension, diabetes mellitus, cardiac dysfunction, chronic kidney failure, stroke, smoking, thalassemia, dyslipidemia, and fibrinogenemia, showed as increased serum D-dimers [10].

The most performed major orthopedic surgeries and often also as the cause of DVT were included in three groups according to the degree of traumatization in the hip and knee joints: Total Arthroplasty (TA) group, Hemiarthroplasty (HA) group, and Internal Fixation (ORIF) group [11].

The dynamics of thrombus formation as the leading cause of extensive DVT in major orthopedic surgeries are still not fully understood. Besides being influenced by the risk factors of elderly patients themselves with various age, gender, body mass index, and various history of the disease, it is also caused by the risk factors of bone surgery due to riches contents of microvascular of the bone marrow, with a variety of etiology, severity, and surgical techniques. Several studies report the term bone debris as a “danger signal” because it contains coagulation activators which could contribute to the processes of hemostasis cascades leading to the formation of DVT, namely collagen, proteoglycan, fibronectin, von Willebrand Factor (vWF), and Tissue Factor (TF) [12], besides P-selectin (Ps) and Nitric Oxide (NO) [13,14].

To study the relationship between the trauma magnitude in the meta-epiphyseal cancellous bone and DVT, five biomarkers were used in the model to represent a pro- or anti-thrombogenic component in the complexity of the hemostasis and inflammation process.

Collagen I (Col I) biomarkers found in 90% of the skeletal bones and a little in the flexible external lamina of blood vessel walls, together with Collagen IV (Col IV) biomarkers found in the basal lamina in the subendothelial matrix of blood vessels are proteins that “do not originate from blood”. They are exposed to bone debris as a result of the traumatization. These Collagens act as triggers (initiator) of platelets in primary hemostasis [15] and also triggers of factor XII in secondary hemostasis [16] at the time of injury.

Tissue factor (TF) biomarkers found in subendothelial tissue, platelets, and white blood cells act as triggers for thrombus formation (initiator), but also as factors that enlarged thrombus nidus (propagator) [17]. In a healthy body balance, an initial blood clot is formed under the injured venous valve [17]. Endothelial cell damage which causes the presence of collagen and TF will activate platelets [18]. This condition will trigger the inflammatory process, as soon as 6 h after thrombus formation, by presenting a Circulating Adhesion Molecule P-selectin (Ps) in the venous wall [12].

P-selectin biomarkers play a role in raising thrombus (propagator) [12], found in platelets and endothelial cells. Biomarkers for thrombus propagation increase for five days and decrease until day 14 [19], with decreasing levels of pro-inflammatory mediators due to a down-regulating mechanism [20].

Nitric Oxide (NO) biomarkers, which are a compound that inhibits platelet adhesion and anti-inflammation, is found in endothelial cells in blood Nitric Oxide (NO) biomarkers, which are a compound that inhibits platelet adhesion and anti-inflammation, is found in endothelial cells in blood vessel walls and in platelets to keep the blood liquid (anti-thrombogenic) [21,22]. NO is a free radical gas molecule that is very reactive and reacts quickly with oxygen forming nitrites and nitrates.

This study aims to reveal the degree of traumatization of meta-epiphyseal cancellous of hip and knee joints in major orthopedic surgery that affects the incidence of DVT through the dynamics of prothrombogenic and anti-thrombogenic of the thrombus formation which involve the role of Col I and Col IV as the initiator of the thrombus formation which originated from the bone and blood vessels; TF and Ps as a thrombus propagator; and NO as antithrombocyte, before surgery, 72 h, and 144 h after surgery. If proven, the biomarker(s) could be used as a predictor that may help in the diagnosis of DVT.

## 2 Patients and methods

This study received ethical approval from the Faculty of Medicine, Hasanuddin University, Makassar, Indonesia with ethical number Ethical approval no 0378/H04.8.4.5.31/PP36-KOMETIK/201 and registered at the research registry, the study also has been reported in line with the STROCSS criteria [32].

This is a clinical observation prospective cohort study. Patients were obtained from outpatient and inpatient care consecutively with subject inclusion criteria: 1) Patients over 50 years of age (all female patients are menopause) who will carry out: elective total arthroplasty surgery for stage III-IV osteoarthritis of the knee or hip joint (TA) group, non-elective hemiarthroplasty surgery for indications of sub-capital femoral fracture of the hip joint (HA) group and non-elective internal fixation surgery for indications of trochanter femur fracture of the hip joint (ORIF) group, 2) Willing to be a participant in the research, after signing volunteerism based informed consent.

With exclusion criteria: 1) History of DVT and pulmonary embolism (PE) history, 2) Using Estrogen as Hormone Replacement Therapy, 3) History of or suffering from malignancies and undergoing chemotherapy, 4) Took anticoagulants/antiplatelets from seven days before surgery, 5) Using thrombo-prophylaxis or elastic compression stockings or graduated compression devices, 6) Total paralysis due to stroke or spinal injury, 7) Using a central venous catheter, 8) Had major surgery in the last three months, 9) History of inflammatory bowel disease and nephrotic syndrome. 10) history hemorrhagic disorder, 11) history of smoking for the last year, 12) sepsis, 13) obese (BMI ≥ 35).

Patients who passed the inclusion criteria were taken for blood sampling of three cc serum of each Col I, Col IV, TF, P-selectin, NO levels before surgery, 72 h, and 144 h after surgery. The laboratory test used for Collagen I is Sandwich Enzyme-Linked Immunosorbent Assay (ELISA) with a standard range of 6.25–400 ng/mL and a detection limit of 0.285 ng/mL. The reagent kit used is USCNK Life Science Inc. Zhuan Yang Avenue with catalog number E90751Hu. Laboratory tests for Collagen IV serum were carried out using the Latex Turbinometric Immunoassay (LTIA) method with an analytical range between 16 and 800 ng/mL with normal values < 140 ng/mL. The reagent brand used was Latex Panassay C.IV IV.C from Daichii Pure Chemical Co. product. Ltd. Tokyo-Japan.

Laboratory tests used for TF were carried out by the colorimetric method with the principle of chromogenic activity. The reagent kit used is the Human Tissue Factor Product Assay Pro MO 63304.The USA. With the Cat catalog number: CT1002b. The standard range is 1.95–500 μm.

Laboratory tests for P-selectin (Ps) were carried out using the direct ELISA method. The reagents used were produced by R&D Systems Minneapolis MN55413 USA with catalog no. BBE6. The standard range is 0.82–46 ng/mL, the detection limit is 0.5 ng/mL.

Laboratory tests for NO were carried out using the colorimetric method. The reagents used were produced by Cayman Ann Arbor, MI 48108 USA with catalog number 780001. The standard range is 5–35 μM, and the detection limit is 2.5 μM.

During treatment, the subject was observed clinically. DVT examination was determined at 144 h after surgery with venographic examination as the gold standard (or Doppler ultrasound if allergic, refuse, or technically not feasible) performed by the same radiologist at each hospital. The venographic examination was performed using the Siremobile compact C-arm according to the Rabinov and Paulin technique.

This study analyses: 1) Demographic description of subjects at the three different operating groups, and biomarker dynamics before surgery, 72 h and 144 h after surgery, as a general description of the research data, 2) The influence of demographic and subject's comorbid on the DVT using Chi-square, to identify factors other than surgery that affect the incidence of DVT, 3) The effect of the degree of the meta-epiphyseal traumatization to the incidence of DVT using Chi-square, to prove that the greater the degree of trauma and the difficulty of the operation, the higher the incidence of DVT by showing the relationship between the three different operating groups and the incidence of DVT, 4) The effect of the degree of traumatization of meta-epiphyseal cancellous on the difference in biomarker levels at 72 h and 144 h after surgery using one-way ANOVA, to prove that the greater the degree of trauma, the greater the presence of pro-thrombogenic agents, 5) The effect of biomarker levels before surgery, 72 h and 144 h after surgery on the incidence of DVT using independent T-test, to prove the incidence of DVT occurs through the role of biomarkers, 6) The ratio of pro-thrombogenic/anti-thrombogenic biomarkers to the incidence of DVT using independent T-test, to prove the dynamics of pro-thrombogenic and anti-thrombogenic interactions with DVT events by showing the relationship of biomarker ratios to DVT events.

Statistical analysis was performed using SPSS version 16.0 for Windows (Chicago, IL, USA) with a significance level of 5% (0.05) for absolute and 10% (0.1) for relative.

## 3 Result

Seventy-four subjects met the inclusion criteria; 69 subjects were studied until the final analysis from May 2010 to September 2011 from two hospitals. Five subjects were drop-out due to two hemolytic serum sample, one patient reject to continue to be in the study, and late findings of exclusion criteria of smoking in one patient and self-administered aspirin in one patient. There were 17 men and 52 women with age >70 years (68.1%). Twenty-three patients were treated with TA, 22 patients treated with HA, 24 patients treated with ORIF. All surgery was performed by the first author, a senior orthopedic consultant. All subjects were treated without thromboprophylaxis (Table 1).

**Table 1:** Patient characteristic based on traumatization degree of meta-epiphyseal cancellous.

Variable	Traumatization degree of meta-epiphyseal cancellous			Total
	TA (n = 23)	HA (n = 22)	ORIF (n = 24)	(n = 69)
Female, n(%)	22 (95,7)	18 (81,8)	12 (50,0)	52 (75,4)
Male, n(%)	1 (5,9)	4 (18,2)	12 (50,0)	17 (24,6)
Age >70, n(%)	11 (47,8)	18 (81,8)	18 (75,0)	47 (68,1)
Age ≤70, n(%)	12 (52,2)	4 (18,2)	6 (25,0)	22 (31,9)
BMI ≥25, n(%)	11 (47,8)	3 (13,6)	6 (25,0)	20 (29,0)
BMI <25, n(%)	12 (52,2)	19 (86,4)	18 (75,0)	49 (71,0)
HT, n(%)	6 (26,1)	12 (54,5)	13 (54,2	31 (44,9)
CKD, n(%)	–	1 (4,5)	1 (4,2)	2 (2,9)
Stroke, n(%)	1 (4,3)	2 (9,1)	5 (20,8)	8 (11,6)
CD, n(%)	1 (4,3)	3 (13,6)	1 (4,2)	5 (7,2)
DM, n(%)	3 (13,0)	6 (27,3)	7 (29,2)	16 (23,2)
Osteoporosis, n(%)	4 (17,4)	15 (68,2)	17 (70,8)	36 (52,2)

TA, Total Arthroplasty; HA, Hemi Arthroplasty; ORIF, *Open Reduction Internal Fixation*; CKD*, Chronic Kidney Disease;* CD, *Cardiac Dysfunction*; DM, Diabetes Mellitus; BMI, Body Mass Index.

Of 69 subjects, 18 subjects have DVT (26.1%), with the distribution of 10 subjects from the TA group (43.5%), five subjects from HA (22.7%), and three subjects from ORIF (12.5%). Women have a greater risk of having DVT (OR = 7.7). It was also found that the diagnosis of OA had a higher risk of DVT compared to a fracture (OR = 3.6), (p = 0.029; p = 0.020). (Table 2).

**Table 2:** The relationship between risk factors and the incidence of DVT.

Characteristic n = 69	DVT n = 18		
	OR	CI 95%	p
Gender (F)	7,77	0,95–63,57	0,029
Diagnose: OA/Fracture	3,65	1,19–11,23	0,020
Age	1,91	0,55–6,67	0,306
BMI	1,32	0,42–4,20	0,636
CKD	1,37	1,18–1,58	1,000^+^
Stroke	0,94	0,17–5,13	0,941
CD	2,00	0,31–13,06	0,600^+^
DM	2,05	0,62–6,80	0,236
Hypertension	1,32	0,45–3,87	0,615
Osteoporosis	0,66	0,22–1,94	0,445

F, Female; OA, Osteoarthrosis; CKD, Chronic Kidney Disease; CD, Cardiac Dysfunction; DM, Diabetes Mellitus; OR, Odds Ratio; CI, Confidence Interval 95%; DVT, Deep Veins Thrombosis; BMI, Body Mass Index; n, number of subjects.

The relationship between the degree of traumatization of meta-epiphyseal cancellous of hip and knee joints in major orthopedic surgery and DVT events is shown by OR of TA:HA: ORIF = 3.5:1.8:1 (Table 3).

**Table 3:** Relationship between traumatization degree of meta-epiphyseal cancellous and DVT.

Trauma Degree	n	DVT		OR		CI 95%	
		Positive n,(%)	Negative n (%)				
TA	23	10 (43,5%)	13 (56,5%)	3,5	1,25	–	23,28
HA	22	5 (22,7%)	17 (77,3%)	1,8	0,43	–	9,87
ORIF	24	3 (12,5%)	21 (87,5%)	1			

TA; Total Arthroplasty; HA, Hemi Arthroplasty; ORIF, Open Reduction Internal Fixation; CI, Confidence Interval 95% n, number of subjects.

Statistical significant differences were found in the dynamics of Col I levels based on the degree traumatization of meta-epiphyseal cancellous (p < 0.05) at the 72 h observation after surgery, where Col I TA levels were higher than HA and ORIF (Table 4). Col I levels and Ps levels before surgery were found to be statistically significant. Col IV levels, TF levels and Ps levels before surgery are lower in TA compared to HA and ORIF with visible TA were lower than HA and ORIF before surgery Col IV levels, TF levels, and Ps levels (Table 4).

**Table 4:** Comparison of dynamics Col I, Col IV, TF, Ps, NO based on traumatization degree of meta-epiphyseal cancellous.

		N	Pre-op (M)	72 h Post-op (M)	144 h Post-op (M)
Col I	TA	23	139,2 (45,60–707,70)^a^	212,50 (82,70–523,20)^a^	198,70 (80,00–734,21)^a^
	HA	22	115,5 (60,78–262,90)^a^	145,55 (59,60–285,80)^b^	119,40 (68,20–310,50)^b^
	ORIF	24	114,69 (61,44–546,00)^a^	164,80 (68,67–457,80)^b^	104,23 (38,35–519,60)^b^
			p = 0,536	p = 0,004	p = 0,002
Col IV	TA	23	115,70 (79,60–193,70)a	150,20 (91,80–452,00)a	155,90 (97,30–261,30)a
	HA	22	161,35 (84,30–272,20)b	174,20 (103,30–477,60)a	174,45 (106,40–356,20)a
	ORIF	24	137,80 (80,40–400,50)b	166,55 (88,40–553,60)a	156,55(100,90–377,40)a
			p = 0,034	p = 0,642	p = 0,638
TF	TA	23	403,80 (184,60–546,20)a	373,00 (160,40–518,40)a	373,00 (234,30–455,00)a
	HA	22	415,40 (289,50–559,00)a	388,65 (88,20–555,10)a	393,05 (148,40–526,40)a
	ORIF	24	355,05(203,80–559,00)b	347,40 (223,00–497,30)a	395,45 (225,90–484,10)a
			p = 0,005	p = 0,289	p = 0,728
Ps	TA	23	75,10 (48,20–162,90)^a^	55,10 (44,70–115,10)^a^	83,90 (47,10–127,80)^a^
	HA	22	85,50 (34,30–154,60)^a^	75,35 (23,10–164,00)^b^	75,00 (40,80–209,40)^a^
	ORIF	24	77,05 (5,10–115,00)^a^	62,80 (13,20–100,70)^b^	93,60 (6,20–153,10)^a^
			p = 0,376	p = 0,076	p = 0,664
NO	TA	23	5,50 (2,49–20,50)^a^	5,70 (2,49–24,30)^a^	4,70 (2,49–19,10)^a^
	HA	22	3,85 (2,49–10,30)^b^	5,00 (2,49–42,80)^a^	4,80 (2,49–25,00)^a^
	ORIF	24	3,45 (92,49–20,80)^b^	4,40 (2,49–13,20)^b^	3,40 (2,49–8,10)^b^
			p = 0,026	p = 0,040	p = 0,080

Col I, Collagen I; Col IV, Collagen IV; TF, Tissue Factor; Ps, P-selectin; NO, Nitric Oxide; M, Median; ^a,b,c^, the same superscript shows no significant difference.

Significant differences were found in the dynamics of NO levels, dynamics of NO in TA were higher than HA and ORIF before surgery (p < 0.05). 72 h after surgery, subjects in TA and HA groups had higher NO levels than the ORIF group and were statistically significant (p < 0.05) with observations until 144 h after surgery, ORIF the group still has lower NO levels than HA and TA (Table 4).

It was found that Col I levels were higher in subjects who experienced DVT at 72 h and 144 h after surgery when compared to those who did not experience DVT. However, it was not statistically significant (p > 0.05) (Table 5). No significant difference was found in Col IV level (p > 0.05). However, there were no significant differences in dynamics after surgery, and the TA group has a lower Col IV level than HA and ORIF before surgery (Table 5). Dynamics of TF levels based on the degree of traumatization, although there were no significant differences in dynamics (p > 0.05) after surgery, it was seen that the HA group has higher TF level than ORIF before surgery, while dynamics of Ps levels based on the degree of traumatization, the HA group pattern is found to slowy decline until 144 h after surgery.

**Table 5:** Comparison of dynamics Col I, Col IV, TF, Ps, NO based on DVT incidence.

	DVT	n	Pre-op (M)	72 h Post-op (M)	144 h Post-op (M)
Col I	Positive	18	131,05 (58,60–707,70)	195,95 (59,60–523,20)	158,71 (68,20–552,50)
	Negative	51	116,00 (45,60–684,48)	171,62 (68,67–510,80)	125,27 (38,35–734,21)
			p = 0,499	p = 0,101	p = 0,243
Col IV	Positive	18	118,30 (90,70–169,60)	167,95 (400,00–362,50)	170,10 (102,90–229,20)
	Negative	51	160,60 (79,60–400,50)	162,40 (91,80–553,60)	163,80 (97,30–377,40)
			p = 0,022	p = 0,891	p = 0,647
TF	Positive	18	399,65 (184,60–559,00)	367,80 (160,40–489,20)	390,60 (252,00–505,40)
	Negative	51	400,20 (203,80–559,00)	363,90 (88,20–555,10)	373,00 (148,40–526,40)
			p = 0,827	p = 0,838	p = 0,558
Ps	Positive	18	73,40 (48,40–162,90)	65,30 (47,50–115,10)	105,00 (47,10–127,80)
	Negative	51	78,30 (5,10–154,60)	63,70 (13,20–164,00)	84,30 (6,20–209,40)
			p = 0,675	p = 0,523	p = 0,271
NO	Positive	18	4,55 (2,49–20,50)	6,65 (2,49–42,80)	4,85 (2,49–25,00)
	Negative	51	4,30 (2,49–20,80)	4,80 (2,49–17,00)	4,10 (2,49–16,80)
			p = 0,325	p = 0,014	p = 0,249

Col I, Collagen I; Col IV, Collagen IV; TF, Tissue Factor; Ps, P-selectin; NO, Nitric Oxide; M, Median.

Significant differences in the dynamics of NO levels were based on the degree of traumatization, where NO levels increased and then decreased again. The dynamics of NO in the TA group were higher than in HA and ORIF group before surgery (p < 0.05). Subjects with TA and HA group at 72 h after surgery had higher levels in the ORIF group and it is statistically significant (p < 0.05). At 144 h after ORIF surgery, they still have lower levels of NO than in HA and TA groups (Table 5).

After comparing the biomarker dynamics based on the degree of trauma and the incidence of DVT, it is seen the significant involvement of biomarkers Col I and NO consistently. Considering that Col I is a pro-thrombogenic initiator and NO is anti-thrombogenic, then a dynamic comparison of Col I/NO ratio is performed based on the degree of traumatization of the meta-epiphyseal cancellous of hip and knee joints in major orthopedic surgery and based on DVT events.

The dynamics of the Col I/NO ratio based on the degree of traumatization the TA, HA, and ORIF groups patterns are different, where the ratio of TA was seen to continue increased up to 144 h after surgery. However, there were no statistically significant differences in dynamics (p > 0.05). While dynamics of the Col I/NO ratio based on DVT events had the level of Col I/NO ratio at 72 h and 144 h was lower when compared to those who did not have DVT. However, there was no statistical difference (p > 0.05) with the incidence of DVT, and there was a tendency for a relationship at 72 h after surgery (p = 0.172) (Table 6).

**Table 6:** Comparison of the dynamics of the Collagen I/Nitric Oxide ratio based on the incidence of DVT.

Ratio Col I/NO				
DVT	N	Pre-op (M)	72 h Post-op (M)	144 h Post-op (M)
Positive	18	27,98 (3,39–284,22)	25,39 (1,39–135,78)	30,71 (2,73–221,89)
Negative	51	26,61 (9,32–207,42)	35,05 (10,10–130,96)	32,12 (9,56–253,18)
		p = 0,924	p = 0,172	p = 0,967

Col I, Collagen I; NO, Nitric Oxide; ^a,b,c^, the same superscript shows no significant difference.

## 4 Discussion

The incidence of DVT in orthopedic surgery is inseparable from the real risk factors in the study subjects (Table 1). The grouping of patients was made based on the degree of traumatization of the meta-epiphyseal cancellous, where the TA group experienced twice as high traumatization degree to the HA group, while the ORIF group with various types of surgery had variations in the degree of traumatization which was certainly smaller than the TA group.

In this study, post-menopausal female patients and OA had interactions with DVT (Table 2). Studies have shown that the risk of DVT in women is higher than in men [23]. Although our study showed similar results, it should be noted that the demographic of our study was also dominated by post-menopausal women (75.4%). The relationship between decreased estrogen with increased microparticles increasing TF production, which led to an increased risk of cardiovascular events [24].

There is no known direct relationship between OA and DVT, but it is known that patients with OA have higher levels of local NO in the joints [24]. High local NO levels in OA patients are caused by the role of NO as a catabolic factor mediating the expression of pro-inflammatory cytokines (IL-1), inhibition of collagen and proteoglycan matrix synthesis, and inducing apoptosis, which is the basis of the pathomechanism of OA. Recent studies have shown the dual role of NO as a catabolic and protective against OA [24]. Therefore, whether OA causes DVT through pro-inflammatory mechanisms needs to be further investigated. The possible association of OA with DVT in this study is due to the patient's distribution in the OA group, which is 95% (22/23 subjects), postmenopausal female.

Major orthopedic surgery is one of the risk factors for DVT due to the traumatization of meta-epiphyseal cancellous hip and knee joints [1]. The results showed that the surgery group with the highest degree of traumatic meta-epiphyseal cancellous had the highest incidence of DVT, as the TA group (43.5%) was higher than HA (22.7%) and ORIF (12.5%) (Table 3). Although the ORIF group seemed more vulnerable and had more risk factors, this group had a lower incidence of DVT compared to the TA group. This proves that the trauma magnitude has more influence on the incidence of DVT. This is because bone surgery induces the activation of intraoperative hemostasis so that the differences in the degree of meta-epiphyseal traumatization will cause different degrees of damage and different rates of thrombotic events. Surgery that minimizes intramedullary femoral pressure during implant cementing, as in total hip replacement, will reduce the presence of bone debris, collagen, or fat, thereby reducing the incidence of DVT and PE [25].

The results of this study were successful in showing that the degree of traumatization based on the surgery group caused gradual differences in the increase in levels in 72 h after surgery for Col I and NO (p = 0.004; p = 0.040) and a tendency towards Ps (p = 0.076). The comparison of the dynamic levels of Col I levels at 72 h after surgery based on the degree of traumatization showed a gradual difference (p = 0.004), where the TA group showed a higher level than HA and ORIF (Table 4). The TA group had a much higher level of difficulty than HA or ORIF.

In total knee replacement (TKR) surgery, five surface areas of the distal femur joint and one surface of the tibial joint are cut. In THR, osteotomy was performed in the femur column, followed by reaming intramedullary metaphysis and diaphysis of the femur and reaming the surface of the acetabulum to the subchondral limit. That means, the TA group performed greater destruction of the meta-epiphyseal cancellous (which is Collagen I) and took a longer surgical duration than HA or ORIF.

Comparison of the dynamics of NO levels at 72 h after surgery based on the degree of traumatization showed a gradual difference (p = 0.040), where the TA group had higher levels than HA and ORIF (Table 4). This occurs because of the mechanism of action and reaction between prothrombogenic Col I and anti-thrombogenic NO. The greater degree of traumatization of the TA group will release greater levels of Col I, resulting in a higher NO level compared to HA or ORIF group.

The results of the analysis of the five biomarkers suggest that there are other factors besides the traumatization of meta-epiphyseal cancellous that affect the dynamics of biomarkers levels. Only Col I was free from confounding factors. On this basis, Col I was used to measuring the degree of traumatization of meta-epiphyseal cancellous at 72 h or 144 h after surgery. (Table 4).

The dynamics of the five biomarkers show that 72 h after surgery is a turning point where the initiator level begins to decrease, which is compensate by the increasing propagator level. Meanwhile, the anti-thrombocyte levels of NO also decreased, compensating the levels of initiator Collagen, but did not compensate for the increase in levels of TF and Ps propagators. The authors suspect that other anti-thrombogenic factors such as anticoagulation factors of heparin-like molecules, thrombomodulin, and TFPI as well as fibrinolytic factors t-PA and plasmin play a role in compensating the levels of thrombus propagator [26]. Thus, it can be presumed that 72 h after surgery could be the starting point of equilibrium. Or, the start of a hypercoagulability pathological condition that can lead to DVT, if the prothrombogenic and anti-thrombogenic homeostasis is not achieved (Table 4).

The pro-thrombogenic serum levels (Col I, Col IV, TF, and Ps) that were measured before surgery, 72 and 144 h after surgery showed only relative significance of Col I at 72 h after surgery with the incidence of DVT (Table 5). While anti-thrombogenic NO levels at 72 h after surgery showed statistical significance with the incidence of DVT.

The statistically less powerful effect of pro-thrombogenic biomarkers (Col I, Col IV, TF, and Ps) with the incidence of DVT may result from the weak role of each biomarker when it stands alone. Another possibility is due to the limited number of samples that cannot show the role of the four biomarkers in the high incidence of DVT in major orthopedic operations.

Analysis of the results showed no relationship between Col I and Col IV with the incidence of DVT. However, it is believed that collagen is one of the primary activators of the thrombus initiator. Collagen through the mechanism of primary hemostasis activates platelets through the Integrin receptor α2β1 and GpVI [16] to form a “platelets plug” [15,27]. Collagen also triggers secondary hemostasis through Factor XII of the intrinsic pathway to produce stable fibrin clots [17,[27], [2][8], [29]]. Thrombus formed due to severe injury to blood vessels reaches 40 times greater when compared to superficial injuries [30].

NO biomarkers successfully demonstrated the DVT incidence due to traumatization of the meta-epiphyseal cancellous hip and knee joints 72 h after surgery (p = 0.014). Traumatization of the meta-epiphyseal cancellous hip and knee joints results in bone debris, known as a “danger signal”, which is the antigen that triggers thrombosis. This condition activates NO, which is a highly reactive gas molecule [31]. NO works as an endogenous inhibitor by inhibiting platelet aggregation and adhesion and also by slowing down the enlargement of the thrombus and works as an anti-inflammatory by preventing monocyte interactions with endothelial cells.

The results showed that patients with DVT had higher NO levels 72 h after surgery compared to non-DVT, which could be explained as an attempt by the body's reaction to prevent excessive thrombus enlargement. Thus, NO in the traumatization of meta-epiphyseal cancellous hip and knee joints can reflect the presence of active thrombus formation or represent strong pro-thrombogenic and anti-thrombogenic activity 72 h after surgery.

Based on the results of the above study, it is believed that the dysregulation of the balance of pro-thrombogenic and anti-thrombogenic factors can cause DVT. Clarifying the distant relationship between biomarkers and the incidence of DVT as a result of traumatization of the meta-epiphyseal cancellous hip and knee joints, a pro-thrombogenic/anti-thrombogenic ratio (Col I/NO) analysis was used. The result shows a less significant relationship (p > 0.172), probably due to the limited number of samples (Table 6).

As a clinical observation prospective consecutive cohort study with a purpose to analyze the complexity of the hemostasis process leading to DVT, this study then met several limitations. First, the study has a limited sample with a variety of risk factors, therefore the option in grouping the subjects is also limited. Second, due to the complex nature of the hemostasis process, there might be other predictor biomarkers that have not been included in this study for analysis.

In future studies, it would be ideal to have groups of more homogenous populations of trauma magnitude, e.g. total hip arthroplasty versus hemiarthroplasty; total knee arthroplasty versus unicompartmental knee arthroplasty; or proximal femur fracture fixation in a similar type and surgical technique.

## 5 Conclusion

The degree of traumatization of the meta-epiphyseal cancellous hip and knee joints affects the incidence of DVT, whose pathomechanism involves an increase in Col I biomarkers as pro-thrombogenic and NO biomarkers as anti-thrombogenic. An estimated 72 h after surgery is a useful period to examine these biomarkers to help predict the diagnose of DVT. Whereas the involvement of other pro-thrombogenic biomarkers of Col IV, TF, and Ps has not yet been proven.

## Ethical approval

Ethical approval no 0378/H04.8.4.5.31/PP36-KOMETIK/2010 from Faculty of Medicine, University Hasanudin, Indonesia.

## Source of funding

The authors declare that sponsors had no such involvement.

## 6 Provenance and peer review

Not commissioned, externally peer-reviewed.

## Consent

Written informed consent was obtained from the patient for publication of this case report and accompanying images. A copy of the written consent is available for review by the editor in chief of this journal on request.

## Author contribution

A contributed to performed the operation, data collection, analysis and interpretation, manuscript drafting, revising, and approval for publishing.

## Registration of research studies

1. Name of the registry: Franky Hartono.

2. Unique Identifying number or registration ID: researchregistry6551.

3. Hyperlink to your specific registration (must be publicly accessible and will be checked): Browse the Registry - Research Registry.

## Guarantor

Franky Hartono.

## Declaration of competing interest

Non declared a conflict of interest regarding the publication of this paper.
